# Early muscle recovery following robotic-assisted unicompartmental knee arthroplasty

**DOI:** 10.1186/s13104-023-06345-8

**Published:** 2023-05-22

**Authors:** Emma Moon, Paul Gaston, James T. Patton, Allison Bell, Philip M Simpson, Gavin J MacPherson, David F. Hamilton

**Affiliations:** 1 Spire Murrayfield Hospital, Edinburgh, UK; 2 Edinburgh Orthopaedics, Royal Infirmary of Edinburgh, Edinburgh, UK; 3 Research Centre for Health, Glasgow Caledonian University, Cowcaddens Road, Glasgow, G4 OBA UK

**Keywords:** Robotics, Robotic-assisted surgery, Unicompartmental knee replacement, Isokinetic dynamometry

## Abstract

**Background:**

Robotic-assisted unicompartmental knee arthroplasty (UKA) improves implant accuracy, however whether this translates to patient function is less clear. Various outcomes have been reported but muscle recovery has not been previously investigated.

**Objective:**

To explore sequential change in lower limb muscle strength following robotic-assisted UKA with isokinetic dynamometry.

**Results:**

12 participants undergoing rUKA for medial compartment osteoarthritis were assessed pre-operatively, and at 6- and 12-weeks post-operatively. Maximal muscle strength changed over time in both quadriceps (p = 0.006) and hamstrings (p = 0.018) muscle groups. Quadriceps strength reduced from 88.52(39.86)Nm to 74.47(27.58)Nm by 6-weeks (p = 0.026), and then recovered to 90.41(38.76)Nm by 12-weeks (p = 0.018). Hamstring strength reduced from 62.45(23.18)Nm to 54.12(20.49)Nm by 6-weeks (p = 0.016), and then recovered to 55.07(17.99)Nm by 12-weeks (p = 0.028). By 12-weeks quadriceps strength was 70% and hamstrings 83% of the values achieved in the un-operated limb. Substantial improvement was seen in all other measures over time, with sequential positive change in Timed-up-and-go test (p = 0.015), 10 m walk test (p = 0.021), range of knee flexion (p = 0.016) and PROMs (p < 0.025).

## Background

Over 100,000 knee arthroplasty procedures are typically performed in the UK each year, of which around 10% are unicompartmental knee arthroplasty (UKA)[1, 2]. Total Knee Arthroplasty has long been considered the gold standard intervention for knee OA due to its demonstrated predictability, durability, and effectiveness [3]. UKA has been suggested to offer increased functional outcomes and faster recovery timelines, however there is an acceptance that this may come at the expense of implant durability, highlighted by higher revision rates [2, 4, 5].

Revision rate is thought to be one of the most important factors for patients and surgeons when it comes to implant choice, and this has likely contributed to generally low rates of UKA uptake in the UK [6]. These poorer long-term outcomes have been variously suggested to result from issues with implant design, bony fixation, surgical instrumentation, surgical technique, component malpositioning and post-operative limb malalignment [7].

Robotic-assisted surgery has demonstrated enhanced implantation accuracy in UKA [8–10], and it is suggested that this improvement in component positioning may contribute to lower revision rates and also to improved functional outcomes through reduced surgical trauma, improved joint kinematics, and enhanced stability and proprioception through optimal soft tissue balancing [11, 12]. The invasiveness of a surgical approach or the extent of soft tissue release performed can affect the magnitude of the local and systemic inflammatory responses, which, in turn, may influence post-operative pain and early functional recovery [11]. Bone resection in robotic-assisted UKA is restricted to the confines of the predefined strereotactic boundaries which may help to reduce peri-articular soft-tissue injury and enhance the patient’s ability to carry out post-operative rehabilitation.

Robotic-assisted arthroplasty though is still in its infancy and detailed clinical outcomes and performance data for these procedures remains comparatively scarce. High levels of early post-operative recovery have been reported using patient reported outcome measures and functional tests [5, 7, 13], however there have been no reports which have specifically evaluated muscle recovery. As such, the aim of this study was to evaluate early muscle recovery in patients undergoing robotic-assisted UKA.

## Methods

This was a single-centre explorative longitudinal cohort to evaluate early muscular recovery following surgery. Ethical approval was obtained from Spire Hospitals review board (review ref: SMH_MAC_Mar2019_EM) prior to commencement of the study and patients were recruited with written informed consent. Patients with isolated medial compartmental osteoarthritis that were scheduled to undergo robotic-assisted uni-compartmental knee replacement at Spire Murrayfield Hospital, Edinburgh, UK, between August 2019 and January 2020 were invited to participate. Isokinetic strength testing was carried out pre-operatively, and then post-operatively at 6- and 12-weeks in a physiotherapy outpatient facility. Additional functional performance assessment and a battery of patient reported outcomes were collected at the same timepoints.

### Surgical intervention

The MAKO robotic-assisted knee system (Stryker, Mahwah, New Jersey) was used in all cases. Tourniquet was not routinely used. Three surgeons experienced in the used of robotic-assisted UKA performed the procedures. A segmented 3D CT scan of the patient’s knee aided surgical planning and component positioning prior to surgery with the aim to optimize bone coverage, restore joint line, minimize bone resection, and correct the mechanical axis. Varus deformity at the knee was corrected passively with manual valgus stress to tension the medial collateral ligament. The correction of the varus deformity was guided by the surgeon’s feel of the soft tissue envelope. The computer virtually positioned the implants and gap values of between 0 and 1.5 mm were deemed acceptable. A cemented Restoris MCK implant (Stryker, Kalamazoo, Michigan) was used in all cases.

### Isokinetic dynamometry

The Biodex System 3 was used for isokinetic strength testing. Peak torque [14] was measured in both quadriceps and hamstrings. An isokinetic testing protocol consisting of flexion/extension, con/con at 60 degrees per second was used following a standardized warm-up comprising 3-minutes on a static exercise bike and low resistance flexion/extension exercises with the dynamometer. The Biodex was set up to test the non-operated leg first, followed by the operated leg, orientation was set at 90 degrees, tilt 0 degrees, and seat orientation 90 degrees. The participant’s knee was aligned to the dynamometer with the knee axis of rotation aligned with dynamometer shaft. The ankle strap was set up proximal to the medial malleolus to ensure full ankle mobility. The participant was stabilized in the chair with shoulder, waist and thigh straps. Participants were asked to push against the lever arm until they hit a block and then to pull back against the lever arm until they hit a block. The patients were informed that the machine would counteract with resistance equal to the force they were applying. Following verbal instruction on the use of the biodex machine, patients were then asked to perform 10 trial repetitions; 5 easy (1–2/10 effort), 5 moderate (4–5/10 effort), these repetitions formed a standardized part of the participants warm-up and allowed the participant to become familiar with the test. Following the warm-up repetitions, the participants were then asked to complete 5x maximal contractions (10/10 effort), interspersed with 10-second rest periods.

### Functional outcomes

The Timed-up-and-go test [15, 16] was used as a measure of function. We assessed the time (in seconds) for an individual to stand up from a chair, walk a distance of 3 m, turn, walk back to the chair, turn, and sit down. A 10-metre walk test was employed as a measure of gait velocity [17]. The participant was asked to walk without assistance for 10 m. Active measurements of flexion and extension were made using universal goniometry as per the protocol described by Jakobsen [18]; supine, with no encumbrance from clothing, using a long-arm goniometer with measurements recorded to 1-degree intervals. The axis of the goniometer was aligned to the center of the lateral femoral condyle. The distal arm was aligned to the lateral malleolus and the proximal arm was aligned with the greater trochanter.

### Patient reported outcomes

We additionally recorded well established patient reported outcomes. The Oxford Knee Score [19] is a well validated tool routinely used to assess the outcomes of arthroplasty scored form 0–48. The Forgotten Joint Score-12 (FJS-12) assess joint awareness during various activities of daily living [20]. Scored 0-100, it is a well validated as a responsive tool in arthroplasty populations [21]. Global knee pain severity was assessed using an 11-point scale (0–10) numerical pain rating scale (NPRS) [22], where 0 represents no pain and 10 represents the worst possible pain. We interpret changes in scores over time in reference to the established minimal clinically important differences (MCID) of the scores: OKS, 4-points [23]; JFS-12, 10.8-points [24]; NPRS, 1.1-points [25].

### Statistical analysis

A conservative approach was adopted for dealing with low participant numbers. Data is described descriptively with mean/median and measures of dispersion as appropriate based on the parametricity of the data and differences over time analyzed with non-parametric statistics. Primary analysis was difference in variables across the 3-timepoints using Friedman’s analysis of variance by rank, with post-hoc pairwise comparisons with Wilcoxon Signed Rank tests. Data was evaluated in SPSS statistics (Version 26). Significance was accepted at p < 0.05.

## Results

In the study window, 30 patients undergoing MAKO unicompartmental knee arthroplasty under the care of 3 specified orthopedic surgeons at Spire Murrayfield Hospital, Edinburgh, UK, were listed for surgery and invited to participate, 12 of which were recruited to the study. 17 patients refused due to the burden of the additional hospital visits and 1 could not be contacted. Of the 12 participants recruited, 2 (17%) were female and 10 (83%) male, the mean age was 66.6 (SD 7.62). Mean weight for the group was 81.17Kg (SD 15.62). 6 (50%) procedures were carried out on the left knee (all non-dominant leg) and 6 (50%) on the right knee (all dominant leg).

### Isokinetic strength

Primary analysis was change over the 3-assessment periods up to 12-weeks post-operation. Maximal muscle strength (peak torque) changed over time in both quadriceps (p = 0.006) and hamstrings (p = 0.018) on the operated limb, with no change observed in strength of the uninvolved limb (Table 1). Post-hoc pairwise comparisons highlight that quadriceps muscle strength reduced from a baseline 88.52Nm to 74.47Nm by 6-weeks post-operation (p = 0.026), and then recovered to 90.41Nm by 12-weeks (p = 0.018). Similarly, hamstring strength reduced from 62.45Nm to 54.12Nm by 6-weeks (p = 0.016), and then recovered to 55.07Nm by 12-weeks (p = 0.028). By 12-weeks quadriceps strength was 70% of the un-operated limb and hamstrings 83%. Scaled by bodyweight, the same pattern of results was seen with a dip in proportional strength at 6-weeks, recovering by 12-weeks post-operation (Table 1).

**Table 1:** Isokinetic data

	Pre-op	6-weeks	12-weeks	Significance*
**Operated limb**				
Torque (ext.), Nm. Mean (SD)	88.52 (39.86)	74.47 (27.58)	90.41 (38.76)	0.006
Torque (flex.), Nm. Mean (SD)	62.45 (23.18)	54.12 (20.49)	55.07 (17.99)	0.018
Torque/BW (ext.), Mean (SD)	108.66 (44.04)	91.03 (27.76)	112.77 (36.43)	0.010
Torque/BW (flex.), Mean (SD)	65.28 (20.92)	56.95 (15.18)	68.63 (15.77)	0.020
**Control limb**				
Torque (ext.), Nm. Mean (SD)	121.48 (43.07)	125.28 (44.75)	129.11 (46.80)	0.368
Torque (flex.), Nm, Mean (SD)	62.45 (23.18)	62.53 (19.93)	65.53 (20.71)	0.651
Torque/BW (ext.), Mean (SD)	150.2 (51.32)	153.03 (51.39)	162.96 (62.89)	0.370
Torque/BW (flex.), Mean (SD)	76.11 (24.61)	76.32 (21.02)	82.26 (22.97)	0.650

Ext = extension, flex. = flexion, BW = body weight. *Friedman’s 2-way ANOVA

### Functional outcomes

There was significant improvement in knee flexion of 9.4 degrees in the operated limb between baseline and 12-week assessments (p = 0.016), with post-hoc evaluation highlighting that most of this change happened between 6 and 12-weeks post-op. There was minimal absolute change in knee extension over time with mean changes of less than 1 degree at repeated measures (p = 0.717). No post-hoc differences were apparent between timepoints (Table 2). Participants timed-up-and-go test results improved significantly by 3.01 s (33%) between baseline ad 12-weeks (p = 0.015). Post-hoc, significant differences were seen between each assessment (p = 0.0 08 and 0.045 respectively). 10 m Walk Test times improved by 1.87 s (23%) over the assessment period (p = 0.021). Post-hoc evaluation highlighted the significant change happened between 6- and 12-week assessments (p = 0.028) (Table 2).

**Table 2:** functional outcome tests

	Pre-op	6-weeks	12-weeks	Significance*
**Functional tests**				
Knee Flexion (deg), Mean (SD)	124.17 (7.33)	126.25 (10.61)	133.47 (8.34)	0.016
Knee Extension (deg), Mean (SD)	0.42 (3.34)	1.27 (3.79)	0.72 (2.56)	0.717
TUG (sec), Mean (SD)	9.03 (2.15)	7.25 (3.42)	6.02 (2.75)	0.015
10 m walk test (sec), Mean (SD)	8.00 (2.18)	7.08 (3.14)	6.13 (2.55)	0.021
**PROMS**				
OKS, Mean (SD)	30.25 (6.90)	37.80 (4.44)	40.55 (5.89)	0.019
FJS-12, Mean (SD)	22.66 (14.75)	45.83 (29.00)	56.44 (35.97)	0.042
NPRS, Mean (SD)	4.00 (2.00)	2.68 (3.10)	1.18 (1.75)	0.004

TUG = timed-up-and-go test, OKS = Oxford Knee Score, FJS-12 = Forgotten Joint Score 12, NRS = Numerical Pain Rating Scale. *Friedman’s 2-way ANOVA

### Patient reported outcomes

Mean Oxford Knee Scores increased by 10.3 points in the assessment window (p = 0.019), a value well above the accepted MCID of 4-points. Post hoc evaluation highlights the major change to be in the initial 6-week period (p = 0.027) which minimal further change between 6 and 12 weeks (p = 0.123). FJS-12 increased by 33.78 points (p = 0.042) between baseline and 12-weeks, substantially more than the reported MCID of 10.8 points. Post-hoc evaluation demonstrated that significant change was seen between baseline and 6-week and 6- and 12-week assessment timepoint (p = 0.016 and p = 0.028 respectively) (Table 2). Pain score (0–10 NPRS) reduced by 2.82 points across the assessment period (p = 0.004), substantially more than the score MCID of 1.1 points. Post-hoc pairwise comparisons show significant differences between baseline and 6-weeks, (p = 0.024), but not between 6- and 12-weeks (p = 0.13) (Table 2).

## Discussion

This explorative work charts the early muscle and functional recovery following robotic-assisted UKA and is the first report of isokinetic lower limb strength data in robotic-assisted UKA. The primary finding is that muscle strength (peak torque) declined at by 6-week assessment compared to baseline scores, but rebounded by the 12-week mark. This change occurred alongside substantial and sequential improvement in functional performance and clinical outcome scores. Patient scores showed significant and clinically meaningful improvements from baseline to 12-weeks assessment. A 33-point improvement in Forgotten Joint Score-12 and 10-point change in Oxford Knee Score was observed in the study window, approximately three times the MCID of these scores [23, 24]. A comparatively well-functioning group prior to surgery was recruited as is typical of UKA cohorts. Mean pre-operative knee flexion was 124 degrees, however this improved by a further 10-degrees in the first 12-weeks post-op. Similarly, timed-up-and-go test times show a sequential improvement over the assessment period.

Knee extensor strength is thought to be an important determinant of physical function following knee replacement, however there is very limited information available regarding muscle strength changes following UKA. Fuchs et al.[26] carried out a cross sectional study, examining 17 patients 21-months post-UKA and 11 healthy controls of comparable age. Even at this prolonged follow-up timepoint deficits of 30% in both quadriceps and hamstrings data was seen compared with the control group. Comparatively, our 12-week data reflects 16% quadriceps and 25% in hamstrings deficits in contrast with that healthy age-matched control group.

## Limitations

The main limitations are the comparatively small sample size (though typical of this type of study) and the single centre cohort design. As such, generalisation to other settings is assumed but unknown. The length of follow-up in this study is also comparatively short at 12-weeks, however this is appropriate as our focus was on the muscle strength and physical function in this early timeframe.

## Data Availability

The datasets used and/or analysed during the current study are available from the corresponding author on reasonable request.

## Abbreviations

UKA: Unicompartmental knee arthroplasty
OKS: Oxford Knee Score
FJS-12: Forgotten Joint Score − 12
NPRS: Numerical Pain Rating Scale
MCID: Minimal Clinically Important Difference
